# Nature and Prevalence of Long-Term Conditions in People With Intellectual Disability, a Study That Combines the Powers of AI, Big Data and Lived experience

**DOI:** 10.1192/bjo.2024.192

**Published:** 2024-08-01

**Authors:** Gemma Lewin, Rania Kousovista, Emeka Abakasanga, Rishika Shivamurthy, Georgina Cosma

**Affiliations:** 1 Leicestershire Partnership Trust, Leicester, United Kingdom; 2 Loughborough University, Computer Science, Loughborough, United Kingdom.; 3 Loughborough University, Loughborough, United Kingdom

## Abstract

**Aims:**

Individuals with intellectual disability (ID) exhibit elevated health needs when compared with the general population. There is a higher vulnerability to long-term conditions. A scoping review identified that individuals with ID exhibit a distinct pattern of multiple long-term conditions (MLTC) that is different to the general population. Findings highlight health challenges faced by individuals with ID, emphasising the need for targeted and early interventions to address their unique healthcare needs.

This study utilises a professional advisory panel (PAP) and patient and public involvement (PPI) group to form a consensus on relevant long-term conditions for people with ID. Machine learning algorithms are employed to identify long-term conditions in a large, population-based data repository covering the whole of Wales revealing a comprehensive range and prevalence of long-term conditions in a sample of 13,361 adults with ID.

**Methods:**

A consensus on relevant long-term conditions for people with ID was formulated through iterative review followed by revision by PAP and PPI group. PAP comprised a multidisciplinary team with relevant expertise including General Practitioners, a Consultant Psychiatrist, nurses, pharmacists, and data analysts. The PAP worked in collaboration with a PPI group, comprising three groups of experts by experience: people with ID, family or informal carers of people with ID, and professional carers of people with ID.

This study utilises machine learning algorithms in the Secure Anonymised Information Linkage (SAIL) databank to identify the range and prevalence of long-term conditions in ID. SAIL is an anonymised, population-based data repository, comprising billions of anonymised records across Wales. This study included 13,361 ID adult patients.

**Results:**

Following iterative review and revision by the PAP and PPI group, a consensus of 40 long-term conditions relevant for people with ID was identified. Prevalence rates for each condition were calculated. Ten most prevalent conditions were recorded as mental illness, reflux disorders, epilepsy, chronic airway diseases, hypertension, thyroid disorders, chronic arthritis, chronic kidney disease, diabetes, and anaemia.

**Conclusion:**

Consensus on relevant long-term conditions for the general population developed through previous studies is not relevant for the ID population. This is the first effort at creating a full range of long-term conditions for individuals with ID, utilising a population-based data repository. It is possible to do this in partnership with PAP and PPI groups. Along with prevalence, impact of ageing and gender, and hospitalisation as outcome data, this study describes challenges associated with interpreting data captured by Read Codes and ICD–10 codes.

